# Burkitt Lymphoma Mimicking Advanced Stage Ovarian Carcinoma

**DOI:** 10.7759/cureus.5411

**Published:** 2019-08-17

**Authors:** Hüseyin Alakuş, Mustafa Göksu, Fadime Tosun, Ayşe Gül Örmeci

**Affiliations:** 1 Surgery, Adıyaman University Training and Research Hospital, Adıyaman, TUR; 2 General Surgery, Adıyaman University Training and Research Hospital, Adıyaman, TUR; 3 Anesthesia and Reanimation, Adıyaman University Training and Research Hospital, Adıyaman, TUR; 4 Pathology, Adıyaman University Training and Research Hospital, Adıyaman, TUR

**Keywords:** ovarian carcinoma, non-hodgkin lymphoma, burkitt lymphoma

## Abstract

Lymphoproliferative disorders presenting with clinical features similar to ovarian tumors are a rare clinical condition. Even though lymphomas of ovarian origin are rare, they should be considered during the differential diagnosis of tumors of ovarian origin. In this case report, we aimed to present a case of Burkitt lymphoma that mimics advanced ovarian carcinoma.

## Introduction

Lymphoproliferative disorders presenting with clinical features similar to ovarian tumors are rarely met, and they may be observed as primarily of ovarian origin or secondary to systemic disorders [1]. Non-Hodgkin’s lymphoma of primary ovarian origin is met quite rarely. It constitutes 0.5% of lymphomas of the genital system and 1.5% of all ovarian-origin tumors [2]. The most commonly observed type is diffuse large B-cell lymphoma [3].

## Case presentation

**Figure 1:**
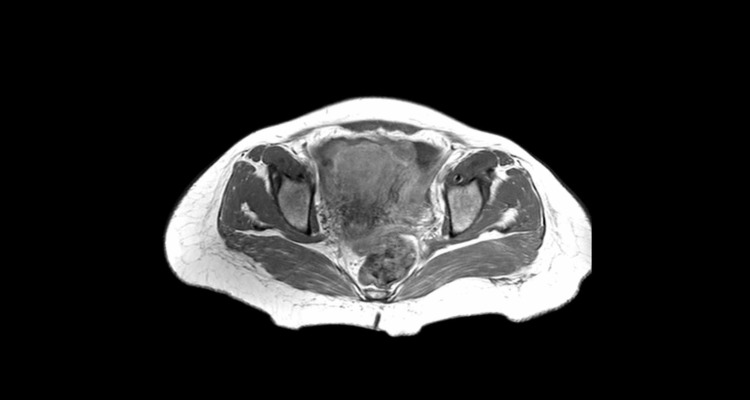
Abdominal MRI image

A 46-year-old female patient presented to the outpatient clinic with the complaints of abdominal distention and constipation lasting for approximately six months. The clinical examination revealed abdominal distention and ascites. In the conducted lower abdominal MR imaging study, the left ovary was 5.5 cm x 3.5 cm in dimension, and solid areas having cystic parts centrally and showing peripheral contrast dyeing were observed (Figure 1).

**Figure 2:**
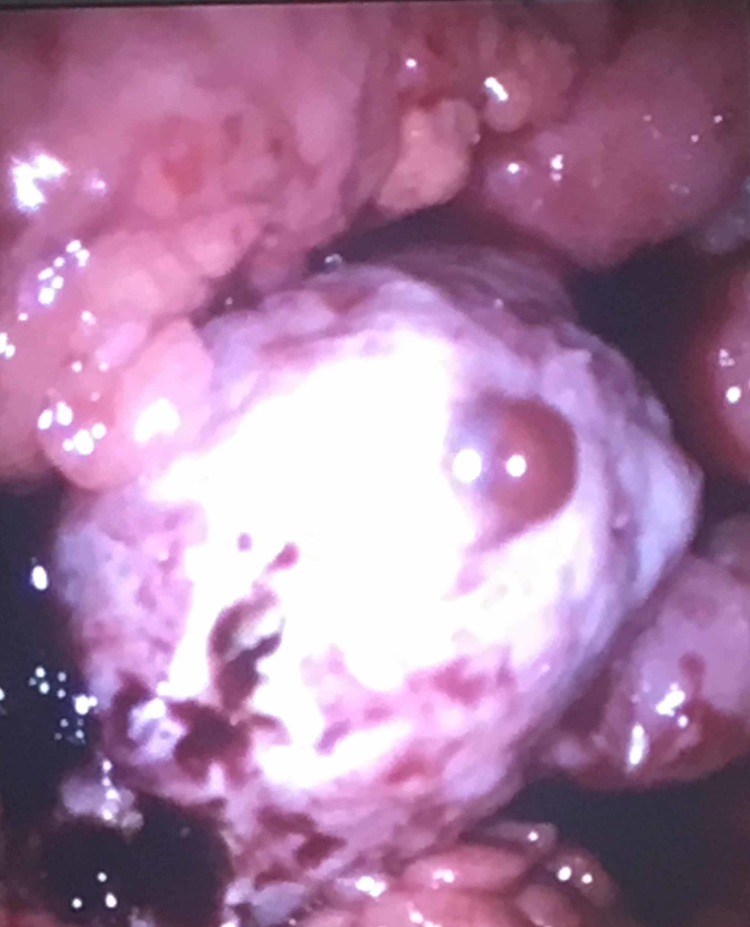
Intraoperative view of the left ovary

An intraabdominal omental cake appearance was present. No pathological lesion was detected in the performed upper and lower GI endoscopies. The positron emission tomography-computed tomography (PET-CT) imaging revealed increased fluorodeoxyglucose (FDG) uptake at the field corresponding the left adnexal site (SUVmax:15.4). Additionally, lesions with increased FDG uptake in the abdominopelvic regions were determined to be consistent with peritonitis carcinomatosis. The laboratory investigation revealed serum lactate dehydrogenase (LDH) level as 404 U/L (normal < 250 U/L), serum albumin level as 3.9 g/dl (normal 3.5-5 gr/dl), serum CA-125 level as 1375.2 U/ml (normal < 35 U/ml). The CA 19-9, CA 15-3, carcinoembryonic antigen (CEA), and alpha-fetoprotein (AFP) levels were determined to be within normal limits. A diagnostic laparoscopy was performed. The exploration revealed an ascites with a volume of approximately 3 L and peritonitis carcinomatosis. The left ovary was approximately 6 cm x 4 cm in dimension. The right ovary was normal. A wedge biopsy of the left ovary was performed together with omental and peritoneal biopsies (Figure 2).

**Figure 3:**
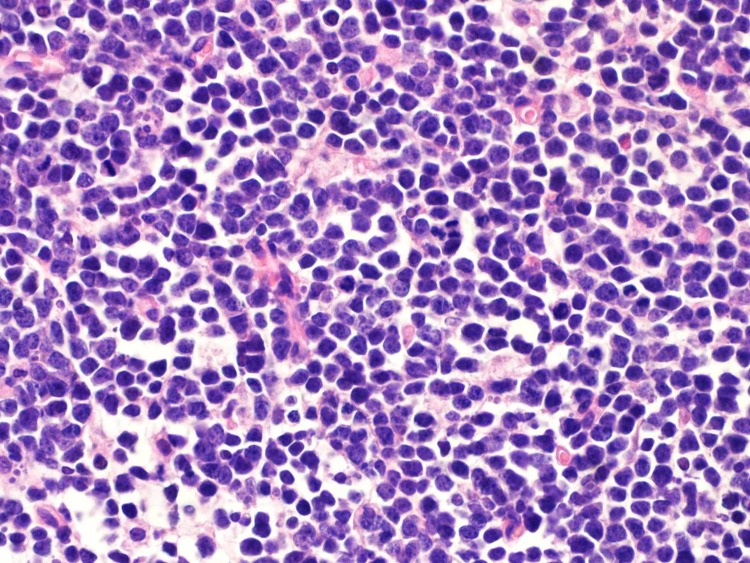
Large cells like immunoblasts showing diffuse growth model

A sample of ascitic fluid was obtained for cytological examination, and the cytopathological examination was reported as positive for the presence of a malignancy. The obtained ovarian, omental, and peritoneal tissues were reported to be tumoral tissues entirely. A tumoral infiltration consisting of uniform, medium-sized, round, lymphocytic cells was observed. The cells had round nuclei, with rough chromatin structure, multiple nucleoli, and narrow cytoplasm, and the mitosis was determined to be present frequently (Figure 3).

**Figure 4:**
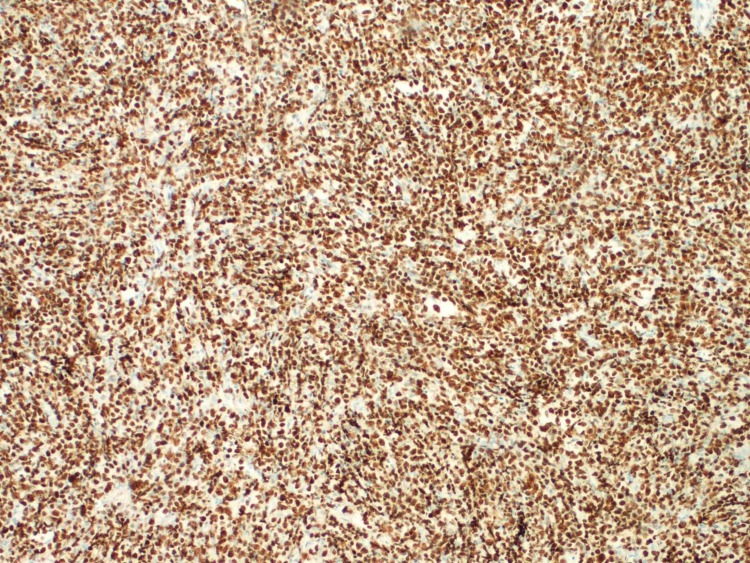
Ki-67 proliferation index

The conducted histochemical studies revealed staining of the tumor cells with LCA, CD20, CD10, CD79a, Vimentin, Bcl6 (poor focally), MUM1 (poor focally), and CD43 (poor focally). Ki-67 index was determined to be 95% (Figure 4).

The pathological diagnosis was reported as a high-grade non-Hodgkin’s B-cell lymphoma. The high proliferation index of the case, together with the histomorphologic and immunohistochemical findings led the patient to be considered to have Burkitt lymphoma on a preferential basis. The patient was transferred to the hematology clinic for treatment.

## Discussion

The ovarian involvement has been reported as 7-26% in lymphomas [4]. They constitute approximately 1.5% of the tumors of ovarian origin [5]. Burkitt lymphoma is even rarer. Non-Hodgkin’s lymphomas constitute approximately 0.5% of the lesions of ovarian origin [6]. Abdominal pain and distention together with nausea are some of the most common symptoms [7]. Our patient had presented with these symptoms also. The most common finding on physical examination is pelvic mass. Bilateral involvement is seen in 40-50% of patients [8]. However, unilateral ovarian involvement was present in our patient. Burkitt lymphoma should be primarily considered in young patients with rapidly progressing bilateral ovarian involvement and elevated LDH levels, because such clinical findings may be easily confused with an epithelial tumor of ovarian origin [9,10]. With this consideration, we planned to perform diagnostic laparoscopy and biopsy initially. The diagnosis is usually made postoperatively with the pathological examination of the specimen in these patients. However, a diagnostic laparoscopy with biopsy, which is a minimally invasive procedure, performed before scheduling surgery can avoid unnecessary radical surgical intervention and surgical stress in these patients.

## Conclusions

As a conclusion, even though lymphomas of ovarian origin are rare, they should be considered during the differential diagnosis of tumors of ovarian origin, and diagnostic laparoscopy and biopsy may be performed.
